# Identification of a Novel Homozygous Nonsense Mutation Confirms the Implication of *GNAT1* in Rod-Cone Dystrophy

**DOI:** 10.1371/journal.pone.0168271

**Published:** 2016-12-15

**Authors:** Cécile Méjécase, Caroline Laurent-Coriat, Claudine Mayer, Olivier Poch, Saddek Mohand-Saïd, Camille Prévot, Aline Antonio, Fiona Boyard, Christel Condroyer, Christelle Michiels, Steven Blanchard, Mélanie Letexier, Jean-Paul Saraiva, José-Alain Sahel, Isabelle Audo, Christina Zeitz

**Affiliations:** 1 Sorbonne Universités, UPMC Univ Paris 06, INSERM, CNRS, Institut de la Vision, Paris, France; 2 CHNO des Quinze-Vingts, DHU Sight Restore, INSERM-DHOS CIC1423, Paris, France; 3 Institut Pasteur, Paris, France; 4 CNRS, UMR 3528, Paris, France; 5 Université Paris Diderot, Sorbonne Paris Cité, Paris, France; 6 Université de Strasbourg CNRS-Icube, UMR 7357, LBGI, Faculté de Médecine, Strasbourg, France; 7 Fondation Ophtalmologique Adolphe de Rothschild, Paris, France; 8 IntegraGen SA, Genopole, Campus, Evry, Paris, France; 9 Institute of Ophthalmology, University College of London, London, United Kingdom; 10 Academie des Sciences, Institut de France, Paris, France; Medizinische Universitat Innsbruck Department fur Kinder- und Jugendheilkunde, AUSTRIA

## Abstract

*GNAT1*, encoding the transducin subunit Gα, is an important element of the phototransduction cascade. Mutations in this gene have been associated with autosomal dominant and autosomal recessive congenital stationary night blindness. Recently, a homozygous truncating *GNAT1* mutation was identified in a patient with late-onset rod-cone dystrophy. After exclusion of mutations in genes underlying progressive inherited retinal disorders, by targeted next generation sequencing, a 32 year-old male sporadic case with severe rod-cone dystrophy and his unaffected parents were investigated by whole exome sequencing. This led to the identification of a homozygous nonsense variant, c.963C>A p.(Cys321*) in *GNAT1*, which was confirmed by Sanger sequencing. The mother was heterozygous for this variant whereas the variant was absent in the father. c.963C>A p.(Cys321*) is predicted to produce a shorter protein that lacks critical sites for the phototransduction cascade. Our work confirms that the phenotype and the mode of inheritance associated with *GNAT1* variants can vary from autosomal dominant, autosomal recessive congenital stationary night blindness to autosomal recessive rod-cone dystrophy.

## Introduction

**Fig 1:**
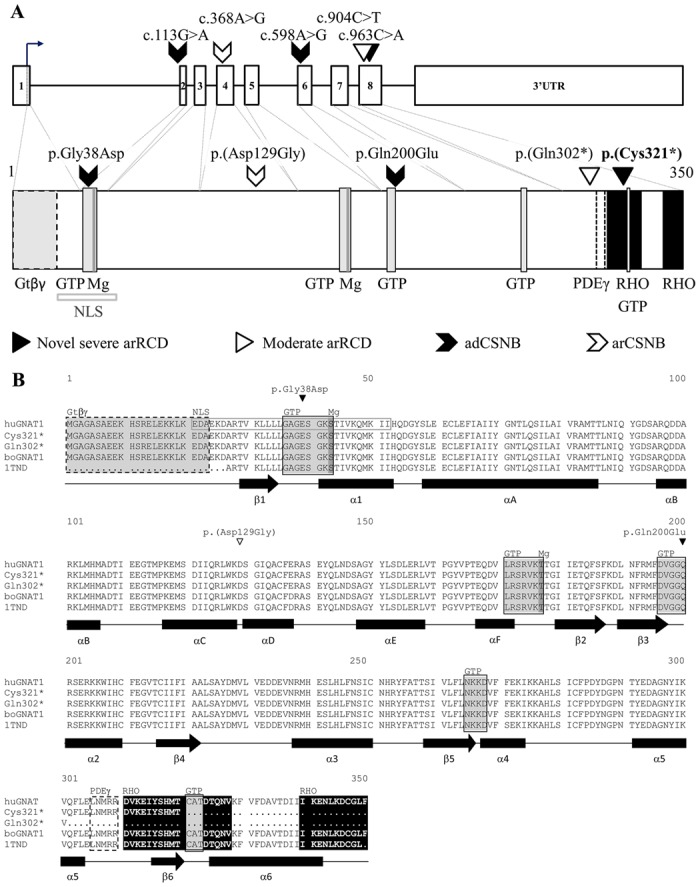
Mutation and protein consequences in *GNAT1*. (A) Known and novel mutations leading to CSNB or RCD on the genomic structure of *GNAT1* (upper part) and the respective protein consequences (lower part). Different arrows indicate the mutation site and associated phenotype. C-terminal nonsense variants were associated with severe RCD (present study) or moderate RCD [8], while missense variants were associated with adCSNB and arCSNB affecting the nuclear localization signal (NLS) and/or GTP/GDP-binding site (GTP) (adCSNB) and an unknown domain of GNAT1 (arCSNB) (lower part) [2–4,9]. (B) The protein is highly conserved in metazoa from human to hydra (data not shown), with 99% of identity between bovine and human GNAT1. Amino acid sequences of the human normal (huGNAT1) and two mutants, (Cys321* and Gln302*) GNAT1, of the bovine GNAT1 (boGNAT1) and the bovine GNAT1 sequence used for crystallization of the protein (1TND) [9]. This last sequence corresponds to the bovine GNAT1 sequence lacking 25 amino acids at the N-terminus and the last phenylalanine amino acid residues, at position 350 [9]. In addition, known CSNB causing mutations are depicted. Human and bovine amino acid sequences are highly conserved. α-helices are represented in black rectangles and β sheets in black arrows (below amino acid sequences) and named as previously reported [9] except for the α-helices G, 4 and 5 which became here 4, 5 and 6, respectively. Specific binding sites are present at following amino acid residues: βγ transducin binding at 1 to 23 (Gtβγ; black dotted and gray shaded box, [9]), NLS at 21–52 (NLS, gray unfilled box, predicted by a software, NLS Mapper), Magnesium binding sites at 43 and 177 (Mg, dark shaded box, predicted by Uniprot, GNAT1_HUMAN), GTP/GDP binding sites at 36–43, 171–177, 196–200, 265–268 and 321–323 (GTP, light grey shaded boxes, predicted by Uniprot, GNAT1_HUMAN and [9]), PDE6γ inhibitory binding site at 306–310 (PDEγ, black dotted unfilled boxes, [9,10]) and activated-RHO binding sites at 311–328 and 340–350 (RHO, black filled boxes, [9]).

The phototransduction is the first step initiating visual signal process within the retina. The rod-specific Gα transducin subunit, encoded by *GNAT1* (Guanine nucleotide-binding protein G(t) subunit alpha-1; MIM#*139330) is a key element of this phototransduction cascade [1]. Defects in *GNAT1* have been identified to cause autosomal dominant and autosomal recessive congenital stationary night blindness CSNB (adCSNB; MIM#610444 [2,3] and arCSNB; MIM#616389 [4]) (Fig 1) showing a Riggs-type electroretinogram (ERG), which is characterized by a- and b-wave amplitude reduction on the dark-adapted ERG responses secondary to rod phototransduction dysfunction [5] and no photoreceptor degeneration. In contrast, ERGs from *Gnat1* null mice also revealed a- and b- wave reduction under scotopic conditions, however with a shortening of rod outer segments and subsequent progressive loss of photoreceptor nuclei [6]. Therefore this mouse model resembles more the phenotype of rod-cone dystrophy (RCD), also known as retinitis pigmentosa (RP; MIM#268000) [7] than CSNB. RCD is a progressive disease, characterized by an initial night vision defect followed by visual field constriction and loss of central vision in severe cases. Recently, a homozygous nonsense variant in *GNAT1*, c.904C>T p.(Gln302*), has been identified in a 80 year-old subject with moderate arRCD [8] (Fig 1) originating from a Irish population.

**Fig 2:**
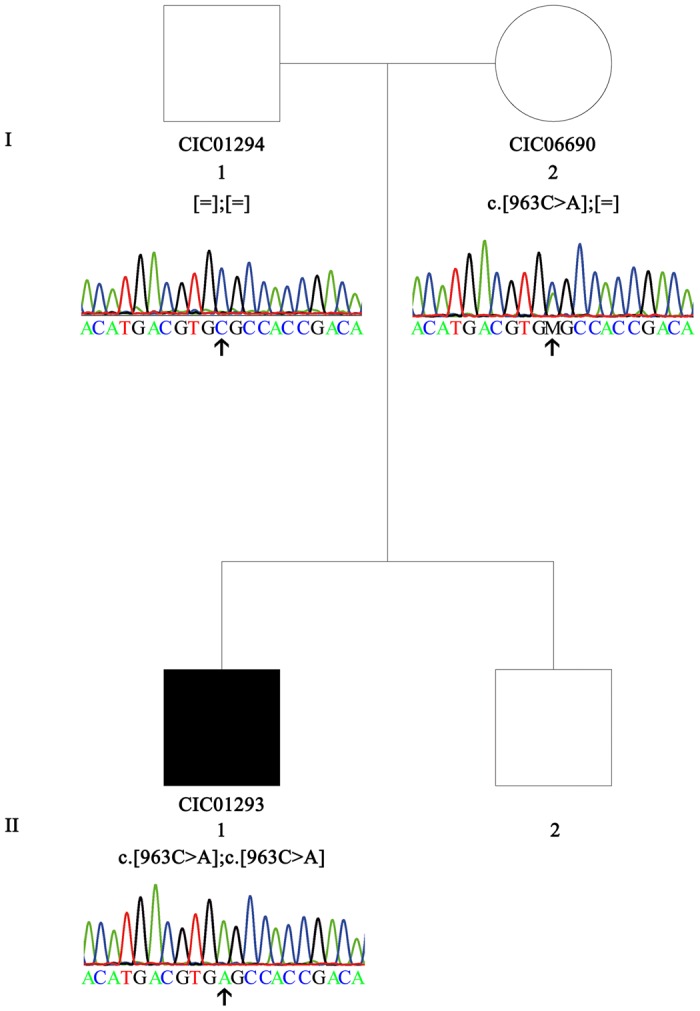
Validation and co-segregation of *GNAT1* variant in family F780. The pedigree and the respective electropherograms of each tested family member are depicted. Family F780 is composed of two unaffected parents (father: I.1, CIC01294; mother: I.2, CIC06690), one affected son (II.1; CIC01293) and one unaffected son (II.2). The nonsense variant c.963C>A p.(Cys321*) [M] in *GNAT1* (NM_144499.2; MIM *139330) was found homozygous in the affected boy (II.1, CIC01293), heterozygous in the unaffected mother (I.2, CIC06690) and absent in the unaffected father (I.1, CIC01294). Females and males are depicted by circles and squares, respectively. Filled and unfilled symbols indicate affected and unaffected status, respectively. The arrow indicates the nucleotide position 963 heterozygously and homozygously changed in the mother and index patient, respectively, and unchanged in the father.

The purpose of our work was to identify, through whole exome sequencing (WES), the gene defect of a 32 year-old male, a sporadic case with RCD diagnosed in teen age (Fig 2). Mutations in 123 genes underlying progressive inherited retinal disorders studied by targeted next generation sequencing (NGS) had previously been excluded [11]. Our study reveals a novel homozygous nonsense variant in *GNAT1* in this severe RCD sporadic case.

## Materials and Methods

### Clinical studies

The sporadic case affected with RCD (Fig 2) was clinically investigated at the national reference center for rare diseases of the Centre Hospitalier National d’Ophtalmologie des Quinze-Vingts. Ophthalmic examination of the proband was performed as previously described [12].

### Targeted next generation sequencing (NGS), whole exome sequencing (WES) and genetic analysis

Blood samples of the index (CIC01293, II.1) and his parents (CIC01294, I.1 and CIC06690, I.2) were collected for genetic research and genomic DNA was extracted as previously reported [13] (Fig 2). Research procedures adhered to the tenets of the Declaration of Helsinki and were approved by the local Ethics Committee (CPP, Ile de France V). Prior to genetic testing, written informed consent, which had been previously approved by the CPP, was obtained from each study participant. Targeted next generation sequencing (NGS) and whole exome sequencing (WES) were performed in collaboration with a company (IntegraGen, Evry, France) [14,15]. A panel of 123 genes known to be associated with retinal dystrophies was used for targeted NGS as previously described [11]. Subsequently, WES was performed in the trio (proband and parents): exons of DNA samples were captured and investigated as shown before with in-solution enrichment methodology (SureSelect Clinical Research Exome, Agilent, Massy, France) and NGS (Illumina HISEQ, Illumina, San Diego, CA, USA) [16]. For all subjects, overall WES coverage of the captured regions was 96% and 90.67% for 10x and 25x depth of coverage respectively resulting in a mean sequencing depth of 82x per base (S1–S3 Tables). Image analysis and base calling were performed with Real Time Analysis software (Illumina) [16]. Genetic variation annotations were realized by an in-house pipeline (IntegraGen), and results were provided per sample or family in tabulated text files. Stringent filtering criteria were used to select most likely pathogenic variant(s): only nonsense, missense, splice site variants or small deletions or insertions (InDels) with a minor allelic frequency ≤0.005 in Exome Variants Server (EVS, http://evs.gs.washington.edu/EVS/), HapMap (http://hapmap.ncbi.nlm.nih.gov/), 1000Genomes (http://www.1000genomes.org/) and Exome Aggregation Consortium (ExAC, http://exac.broadinstitute.org/) were considered to be putative disease-causing. Variant pathogenicity was predicted with bioinformatic tools: Polymorphism Phenotyping v2 (PolyPhen2, http://genetics.bwh.harvard.edu/pph2/), Sorting Intolerant From Tolerant (SIFT, http://sift.jcvi.org/), MutationTaster (http://www.mutationtaster.org/) and amino acid conservation across species was studied with UCSC Genome Browser (http://genome.ucsc.edu/index.html; Human GRCh37/hg19 Assembly).

The *GNAT1* variant selected after WES was validated in the index case and the unaffected parents as following: 2 ng of Genomic DNA was amplified by polymerase chain reaction using oligonucleotides (human *GNAT1* reference sequence NM_144499.2, Forward: 5’-GAGCCCAGAGAGCAGGTG-3' and Reverse: 5’-GGAGCTGGACGGGGCTG-3') (Sigma, Saint Quentin Fallavier, France) at a concentration of 10 μM each with 1.5 mM MgCl_2_ (Solis BioDyne, Tartu, Estonie), 3x S solution (Solis BioDyne), 1x B2 buffer (Solis BioDyne), 0.004 U of a polymerase (Hot Fire Polymerase, Solis BioDyne) and 0.2 mM dNTPs (Solis BioDyne) with an annealing temperature of 60°C and as previously described [11]. The novel *GNAT1* nonsense variant identified in this study has been deposited in dbSNP database (https://www.ncbi.nlm.nih.gov/snp) prior to publication.

A cohort of additional 384 probands with RCD was studied by targeted NGS, including *GNAT1* as previously described with an updated panel covering 195 different genes implicated in RCDs [11,15].

### Tridimensional structure of GNAT1

To identify GNAT1 proteins previously crystallized, BLAST searches (protein Basic Local Alignment Search Tool, BLAST, http://blast.ncbi.nlm.nih.gov/Blast.cgi) were performed against the Protein Data Bank (PDB, http://www.rcsb.org/pdb/home/home.do) using the GNAT1 protein sequence (human GNAT1 reference sequence NP_653082.1) as a query. The sequences (Bovine NP_851365.1 GNAT1, human, wild-type and the two RCD mutant forms from NP_653082.1, and the crystallized bovine GNAT1 (1TND, PDB ID)) were then aligned using Clustal Omega (http://www.ebi.ac.uk/Tools/msa/clustalo/). The three GNAT1 3D-structures, the full-length and the two truncated mutants of NP_653082.1, were predicted (Protein Homology/analogY Recognition Engine V2.0, Phyre^2^, http://www.sbg.bio.ic.ac.uk/phyre2/html/page.cgi?id=index, [17]; Iterative Threading ASSEmbly Refinement, I-TASSER, http://zhanglab.ccmb.med.umich.edu/I-TASSER/, [18]; TM-Align, http://zhanglab.ccmb.med.umich.edu/TM-align/, [19]). The PyMOL Molecular Graphics System, Version 1.7.x Schrödinger, LLC, was used to model GNAT1 interactions with GDP (PDB code 1GOT), GTP (PDB code 1TND), and with RHO (PDB code 4A4M).

## Results and Discussion

### A sporadic case with severe arRCD

**Fig 3:**
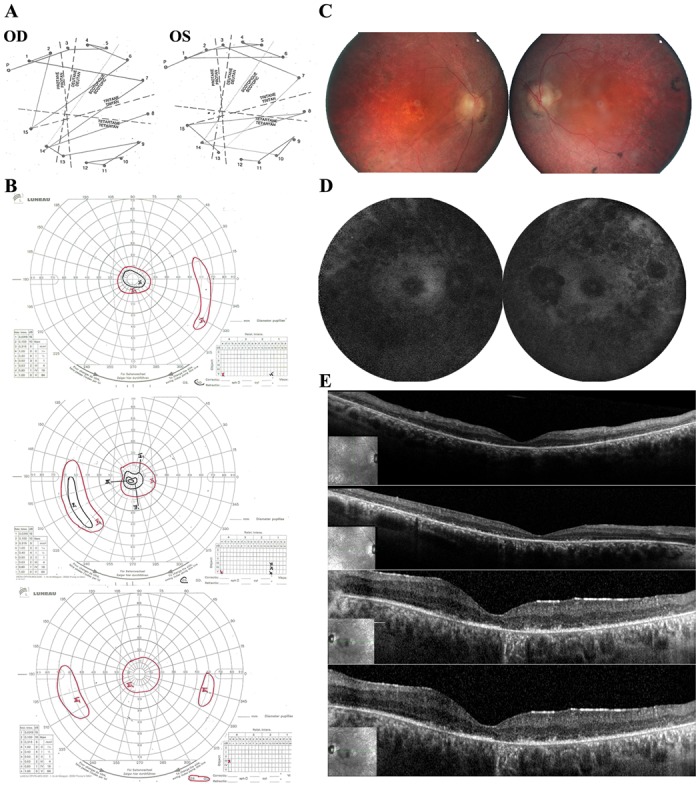
Clinical observations. (A) Color vision test with the Farnworth desaturated 15HUE shows a tritan axis defect. (B) Kinetic visual field tests demonstrate visual field constriction in both eyes. (C) Color fundus photographs reveal optic nerve pallor, narrowed retinal vessels, pigment clumping in retinal periphery some of which resembling more to coarse nummular pigments rather than classical bone spicules, as well as perifoveal atrophic changes. (D) Short-wavelength fundus autofluorescence shows hypo-autofluorescence in the periphery as well as in the perifoveal area. In (A, C, D), Ocula dextra (right eye; OD) is presented in the left part and ocular sinistra (left eye; OS) in the right part. (E) SD-OCT reveals thinning of the outer retinal layers. The two first SD-OCT correspond to OD results, the two next to OS results.

The proband CIC01293 (Fig 2) was a 32 year-old male subject at the time of examination. He had been diagnosed with RCD in his mid-teens secondary to night vision disturbances and progressive visual field constriction. He had no relevant personal medical history. Similarly, there was no familial history of night blindness or retinal disease besides high myopia on father side. He was originating from the south of France with a mother of Italian descent. Best corrected visual acuity was 20/80 with -9(-1)160° in the right eye and 20/63 with -6(-1.50)20° on the left. Color vision with desaturated Farnworth 15Hue revealed a tritan axis defect in both eyes (Fig 3A). Kinetic visual field tests showed bilateral abnormalities with a relative preservation of the central 30° with the III4e stimulus (Fig 3B). Full field ERG was undetectable for both scotopic and photopic responses in keeping with severe rod-cone dysfunction (data not shown). Similarly, multifocal ERG responses were undetectable. Fundus examination revealed optic nerve pallor, narrowed retinal vessels, pigment clumping in retinal periphery some of which resembling more to coarse numular pigment rather than classical bone spicules, as well as perifoveal atrophic changes (Fig 3C). In keeping with fundus changes short-wavelength fundus autofluorescence imaging revealed hypo-autofluorescent lesions in the periphery as well as in the perifoveal area (Fig 3D). Spectral Domain Optical Coherence Tomography (SD-OCT) revealed thinning of the outer retinal layers (Fig 3E). Altogether, these findings were in keeping with severe RCD with macular atrophy. Ophthalmic reports from other family members, including mother, father and brother, did not reveal any night blindness and mentioned normal fundus examination.

### A novel homozygous nonsense variant in *GNAT1* in RCD

Targeted next generation sequencing (NGS) of the coding exons from 123 genes known to be associated with progressive retinal diseases [11,15] applied to the proband's DNA (Fig 2, CIC01293, II.1) did not show pathogenic variants. WES performed in the proband and his unaffected parents (Fig 2, CIC01294, I.1 and CIC06690, I.2) revealed 54.125 single nucleotide variants (SNVs) and 4.009 insertion/deletions (InDels). When considering the autosomal recessive mode of inheritance with SNVs and InDels to be compound heterozygous or homozygous in the affected case and heterozygous in the unaffected parents, no genetic disease variant could be identified. Subsequently, we explored the possibility of *de novo* mutations which have been suggested to play a significant role in sporadic cases with RCD [20] or paternity exclusion. Thus, we investigated only predicted disease-causing variant(s) at the homozygous or compound heterozygous state in the index patient and identified 35 homozygous and 10 compound heterozygous variants and InDels (S4 and S5 Tables), including a homozygous nonsense mutation in exon 8 in *GNAT1*, c.963C>A p.(Cys321*) (A reads 101 times *versus* C reads 2 times; the other nucleotides were not read) (Fig 2). It was rare at the heterozygous state in the general population (ExAC: 0.0000083; 1/119880 alleles) and not reported as homozygous. Since this p.(Cys321*) protein change was predicted to lead to a premature stop codon producing a 29-amino-acid shorter protein, on a gene already implicated in retinal disorders and coding for a protein implicated in the phototransduction cascade, we considered it as the most likely disease-causing variant. Direct sequencing confirmed this variant to be presumably homozygously present in the index patient and heterozygously present in the mother. However, the father did not carry this variant, after testing father's distinct DNA, extracted in two separate sessions as well as an additional sample from a new bleeding (Fig 2). To investigate if the index patient and the father carry a heterozygous exonic deletion missed by Sanger sequencing, we analyzed the coverage of exon 8 (S1 Methods) and did not observe any copy number variation. Interestingly, all 35 homozygous SNVs, including the *GNAT1* variant, detected in the affected proband correspond to the reference allele in the unaffected father. None of the variants identified in index were found heterozygously in the unaffected father (not shown). Supporting this data, without filtering, 3.198 homozygous SNVs and InDels present in the autosomes were homozygous mutated in the affected boy whereas they were homozygous for the reference in the unaffected father (three examples S6 Table). In contrast, only seven SNVs and InDels were homozygous mutated in the affected boy and homozygous for the reference in the unaffected mother. Three additional markers on the Y chromosome were genetically divergent between the father and affected son (S6 Table). Together these results may suggest non-paternity. Further haplotyping was not performed in absence of consent for such a test. Due to numerous probably disease-causing homozygous variants found in the affected boy, homozygosity mapping was performed using the WES data from the affected boy and the unaffected mother to identify large homozygous regions of more than 30 Mb (S2 Methods) and reported in S7 Table. Some homozygous variants identified in the affected boy after filtering were in large homozygous region (S4 Table): interestingly, the *GNAT1* nonsense variant is localized in a large homozygous region (43,6 Mb; S4 and S7 Tables), supporting its involvement in the disease. Additional 384 probands, a majority of which being from European and North African descent, with arRCD were studied by targeted NGS, including *GNAT1* in the panel, and none showed putative pathogenic variants in *GNAT1*. Therefore, mutations in this gene would account for 0.26% of arRCD.

Mutations in *GNAT1*, encoding the rod-specific Gα subunit, were reported in two adCSNB families (Nougaret family (p.Gly38Asp) and p.Gln200Glu, [2,3]) and in one arCNSB family (p.(Asp129Gly) [4]) (Fig 1). All presented a Riggs-type ERG phenotype with a normal fundus appearance, a decreased a-wave amplitude with a corresponding reduced b-wave under scotopic conditions. Interestingly, one subject of the large family with adCSNB carrying the p.Gln200Glu showed RCD at an unreported age, while the 57 year-old daughter did not show any sign of retinal degeneration [3]. Thus it was concluded that RCD was a coincidental finding in this adCSNB pedigree. No further genetic study was performed to identify the underlying genetic defect in this RCD patient. Another hypothesis would be the existence of modifying factors that may have led to retinal degeneration instead of a stationary disease. Longitudinal follow-up is needed in this context to document the stable nature of the disorder. Interestingly, the *Gnat1*^*-/-*^ mouse model shows as well rod photoreceptor dysfunction with late onset degeneration at 51 weeks [6]. Consistent with this model, the first novel nonsense GNAT1 variant, p.(Gln302*), was recently reported in a 80 year-old man with moderate arRCD and predicted to lead to a 48-amino-acid shorter GNAT1 protein [8]. Thus our findings add a second and novel homozygous nonsense mutation to *GNAT1* leading to RCD and support the involvement of *GNAT1* mutations in retinal degeneration. Of note, both truncated GNAT1 proteins due to nonsense variants affected the C-terminal part of GNAT1 are associated with arRCD, whereas missense changes lead to CSNB. Together, these findings suggest that mutations in *GNAT1* can lead to adCSNB, arCSNB and arRCD with variability in phenotypic severity. These findings are important for panel-based targeted NGS, WES or whole genome sequencing approaches investigating progressive inherited retinal disorders. *GNAT1* should be considered as a candidate gene potentially mutated in these progressive disorders: although this remains a rare event with an estimated prevalence of 0.53% [8] and 0.26% in this study.

### Pathogenic mechanism leading to CSNB or RCD

Missense variants that were initially reported underlying adCSNB and arCSNB affect the predicted NLS, the Mg-binding and GDP/GTP-binding domains (Fig 1) [2–4,9]. Pathogenic mechanisms associated with *GNAT1* mutations in adCSNB have been studied in details and mutant-specific mechanisms have been suggested (see for review [5]). In brief, functional *in vitro* assays for the p.Gly38Asp mutant revealed the inability of the activated mutant GNAT1 to bind to the γ subunit of PDE6 and activate PDE6 [21]. These findings were confirmed by biochemical studies on the transgenic mouse model carrying the p.Gly38Asp mutation [22]. In contrast, constitutive activation has been suggested for another transgenic mouse model harboring the p.Gln200Leu GNAT1 variant [23] with a constitutively active α subunit of transducin deficient in GTPase activity. A similar mechanism has been advocated for the p.Gln200Glu variant identified in human adCSNB [3]. Indeed, both mutations affect GTP/GDP-binding domains. The underlying pathogenic mechanism associated with the third GNAT1 mutant, p.(Asp129Gly), identified in arCSNB remains unclear [4]. The mutation is predicted to modify hydrogen bonding to the surrounding amino acid and may induce structural abnormalities important for the proper function of the protein. Further studies are however needed to establish whether this mutant induces a constitutively active protein or a loss of function. The later would fit better with the autosomal recessive mode of inheritance.

**Fig 4:**
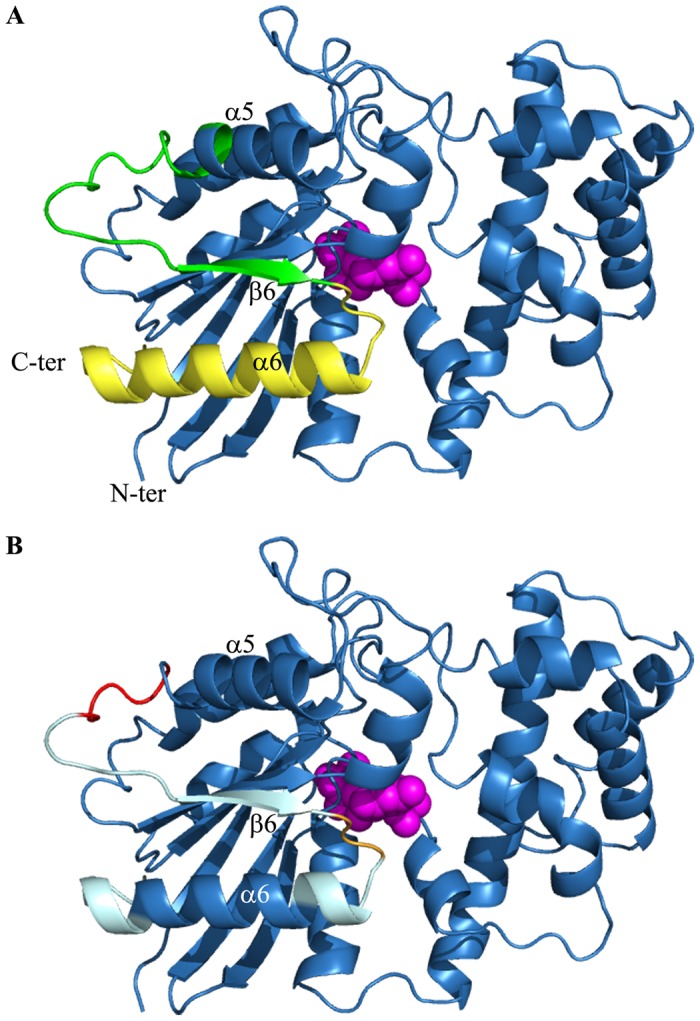
3D-model of normal and mutant GNAT1 interacting with GTPγS. (A) Nonsense variants are predicted to create a shorter protein, compared to normal GNAT1. The p.(Gln302*) mutant loses the last α-helix, α6, (in yellow) and the C-terminal (C-ter) part of the α5-helix and the β6-strand (in green), while the p.(Cys321*) mutant is predicted to keep the α5-helix and the β6-strand (in green) (PDB code 1TND). (B) The C-terminus of GNAT1 is characterized by GTP/GDP (in orange), RHO (in cyan) and PDE6G (in red) binding sites. GTPγS is presented in pink. This protein sequence corresponds to bovine GNAT1 sequence without the 25 amino acid at the N-terminus (N-ter) and the last phenylalanine amino acid, at position 350, residues [9].

The two nonsense *GNAT1* variants associated with arRCD are localized in the last coding exon. It is widely accepted that the last coding exon and the 50–55 nucleotides upstream the last exon-exon junction are NMD-insensitive and therefore mRNA decay in the last coding exon is not a common mechanism [24–26]. The two mutated mRNA are therefore likely to escape nonsense-mediated decay and lead to the production of truncated proteins. The effect of the truncation in the p.(Cys321*) variant on the 3D-structure of GNAT1 was then investigated and compared to the 3D-structure of the wild-type and the previously reported p.(Gln302*) mutant leading to moderate RCD [8]. The 3D-structures were modeled for the wild-type and the two RCD mutants, when the GNAT1 protein interacts with GTPγS (Fig 4; GTPγS in pink) or with GDP (not shown). The global predicted 3D-structures of GNAT1 and the two nonsense mutants are very similar (not shown) [17–19]. While GNAT1 is a 350 amino acid protein, the two mutants are characterized by a shorter protein at their C-terminal end, where 48 and 29 amino acid residues are deleted respectively. The structural differences are indeed localized in this region: the p.(Gln302*) mutant loses the last α-helix, α6, (Fig 4A; in yellow) and the C-terminal part of the α5-helix and the β6-strand (Fig 4A; in green) [8], while the p.(Cys321*) mutant, which is 19 amino acid residues longer compared to the previously reported RCD mutant, is predicted to keep the α5-helix and the β6-strand (Fig 4A; in green). The C-terminal part of GNAT1 is involved in the interactions with GTP/GDP, rhodopsin (RHO; MIM#*180380) and γ subunit of the phosphodiesterase 6 (PDE6G, MIM#*180073) (Fig 4B). The guanine binding site formed by amino acid residues 321 to 323, with Thr323 residue crucial for the binding of the guanine ring, is similarly affected in both mutants (Fig 4B; in orange). Consistent with this finding, an independent *in vitro* study noted that the p.Gln326* mutant is no longer able to bind GTP/GDP [27]. This suggests that the two mutants implicated in arRCD may be unable to interact with or display a decreased affinity for GTP/GDP. Thus, truncated GNAT1 mutants cannot be in the active (GNAT1-GTP) or inactive (GNAT1-GDP) state but could stay in a “nucleotide-free” form [28,29]. This state corresponds to a dynamic intermediate state where GNAT1 interacts with metarhodopsin-II, a RHO intermediate state [30], before the GTP-binding state and transducin activation [28]. An additional *in vitro* study showed that the truncating p.Lys345* GNAT1 mutant loses its ability to bind GTP and RHO, in presence of GTPγS [31]. Albeit many similarities between the two mutants, some interacting domains remain only in the p.(Cys321*) mutant protein compared to the p.(Gln302*) mutant. One of the activated RHO-binding sites, composed by amino acid residues 311 to 328, forming the β6-strand and the α6-helix, is partially impacted by the p.(Cys321*) mutant (Fig 4). The first reported RCD mutant p.(Gln302*) may be unable to interact with RHO, in a “nucleotide-free” form, in contrast to our severe RCD mutant p.(Cys321*) (not tested). This may account for the more severe phenotype of our patient due to an unknown mechanism. Moreover, while the amino acid residues 306 to 310 involved in PDE6 activation are preserved in the p.(Cys321*) mutant (Fig 4), it is not clear if PDE6G can still interact with the “nucleotide-free” GNAT1 form [9,10,32]. Of note, we carefully checked all heterozygous variants that would have acted as modifier factors for disease severity on other genes (123 genes and WES data) previously associated with retinal dystrophies and did not identify additional probably disease-causing mutations: only an homozygous variant c.3376G>A rs144751738 p.(Ala1126Thr) in *RBPP3*, predicted to be tolerated, was observed in the affected boy. Further functional assays are needed to better document this hypothesis. Indeed, a better knowledge of disease mechanism(s) associated with this rod-specifically expressed protein may also have an impact on future therapies.

## Supporting Information

S1 Methods Copy number variation (CNV) analysis. We developed an algorithm to extract CNVs from exome depth of coverage obtained from WES raw data after genomic alignment (i.e. BAM files). In brief, depth of coverage data from the sample to be analyzed were compiled and correlated with a reference depth of coverage dataset obtained from the same sequencing pool with the same targeted NGS and WES strategy. Validation for comparison is made if the correlation coefficient is >0.97. Individual depth of coverage is compared to the depth of coverage reference set for each sample and each target and a score is generated based on the presumed number of copies within the targeted region. Targets with a score ≤ 0.5 (suspected of deletion) or ≥1.5 (suspected of duplication) are selected and subsequently confronted to data from the general population reported in CNV databases (e.g. Database of Genomic Variants, http://dgv.tcag.ca/dgv/app/about) to exclude common variants (>0.005 for recessive variants).
(DOCX)

S2 Methods Homozygosity mapping from WES data. To highlight homozygous regions in rare recessive disorders, an in-house script, developed by a company (Integragen, Evry, France), was used from WES data. Indeed, the method takes advantage of the fact that affected individuals are likely to have two recessive copies of the disease allele from common ancestor alleles.
(DOCX)

S1 Table Coverage and read depth from whole exome sequencing for the affected boy, CIC01293. (DOCX)

S2 Table Coverage and read depth from whole exome sequencing for unaffected mother, CIC06690. (DOCX)

S3 Table Coverage and read depth from whole exome sequencing for unaffected father, CIC01294. (DOCX)

S4 Table Variant found homozygous in the affected boy, CIC01293. ^1^Variant nomenclature was determined with a software (Alamut v2.4, Interactive Biosoftware, Rouen, France). ^2^Conservation: “Highly” means that the same amino acid is conserved in 100 species; “Moderately” means that the amino acid residue varies less than 5 times among species at this position but is conserved in primates; “Weakly” means the amino acid residue varies between 5 to 7 times; if the amino acid residue varies more than 7 times it is qualified as “Not conserved”. ^#^ means that the amino acid residue at the same position changes among primates, but not necessarily with the same amino acid change as the one found in the patient. ^3^ExAC gives the minor allele frequency in a large population from various ethnicities. “Unknown” means that the given change has not been reported in ExAC database. ^4^Retinal expression is determined using UniGene results (http://www.ncbi.nlm.nih.gov/unigene). ^5^Protein domains and splice effect predictions (Alamut v2.4, Interactive Biosoftware; MaxEntScan, http://genes.mit.edu/burgelab/maxent/Xmaxentscan_scoreseq.html, [33]; Splice Site Prediction by Neural Network, NNSPLICE, http://www.fruitfly.org/seq_tools/splice.html, [34]; Human Splicing Finder v.2.4.1, HSF, http://www.umd.be/HSF/#, [35]). G protein-coupled receptor, GPCR; seven-transmembrane domains, 7TM; immunoglobulin-like, plexins, transcription factors domain, IPT/TIG; flavin adenine dinucleotide, FAD.
(DOCX)

S5 Table Variant found heterozygous in the affected boy, CIC01293. ^1^Variant nomenclature was determined with a software (Alamut v2.4, Interactive Biosoftware, Rouen, France). ^2^Conservation: “Highly” means that the same amino acid is conserved in 100 species; “Moderately” means that the amino acid residue varies less than 5 times among species at this position but is conserved in primates; “Weakly” means the amino acid residue varies between 5 to 7 times; if the amino acid residue varies more than 7 times it is qualified as “Not conserved”. ^#^ means that the amino acid residue at the same position changes among primates, but not necessarily with the same amino acid change as the one found in the patient. ^3^ExAC gives the minor allele frequency in a large population from various ethnicities. “Unknown” means that the given change has not been reported in ExAC database. ^4^Retinal expression is determined using UniGene results (http://www.ncbi.nlm.nih.gov/unigene). ^5^Protein domains and splice effect predictions (Alamut v2.4, Interactive Biosoftware; MaxEntScan, http://genes.mit.edu/burgelab/maxent/Xmaxentscan_scoreseq.html, [33]; Splice Site Prediction by Neural Network, NNSPLICE, http://www.fruitfly.org/seq_tools/splice.html, [34]; Human Splicing Finder v.2.4.1, HSF, http://www.umd.be/HSF/#, [35]).
(DOCX)

S6 Table Genotype of the different family members for three genetic markers. (DOCX)

S7 Table Large (>30 Mb) homozygous regions found in the affected boy. Bold large homozygous region includes *GNAT1* variant c.923C>A p.(Cys321*).
(DOCX)
