# Supplementary material for: Identification of a Novel Homozygous Nonsense Mutation Confirms the Implication of GNAT1 in Rod-Cone Dystrophy
Source: PLoS One. 2016 Dec 15;11(12):e0168271. doi: 10.1371/journal.pone.0168271 (PMC5158031; doi:10.1371/journal.pone.0168271)
Supplement: S1 Methods — We developed an algorithm to extract CNVs from exome depth of coverage obtained from WES raw data after genomic alignment (i.e. BAM files). In brief, depth of coverage data from the sample to be analyzed were compiled and correlated with a reference depth of coverage dataset obtained from the same sequencing pool with the same targeted NGS and WES strategy. Validation for comparison is made if the correlation coefficient is >0.97. Individual depth of coverage is compared to the depth of coverage reference set for each sample and each target and a score is generated based on the presumed number of copies within the targeted region. Targets with a score ≤ 0.5 (suspected of deletion) or ≥1.5 (suspected of duplication) are selected and subsequently confronted to data from the general population reported in CNV databases (e.g. Database of Genomic Variants, http://dgv.tcag.ca/dgv/app/about) to exclude common variants (>0.005 for recessive variants). (DOCX) [file pone.0168271.s001.docx]

**S1 Methods: Copy number variation (CNV) analysis**

We developed an algorithm to extract CNVs from exome depth of coverage obtained from WES raw data after genomic alignment (i.e. BAM files). In brief, depth of coverage data from the sample to be analyzed were compiled and correlated with a reference depth of coverage dataset obtained from the same sequencing pool with the same targeted NGS and WES strategy. Validation for comparison is made if the correlation coefficient is >0.97. Individual depth of coverage is compared to the depth of coverage reference set for each sample and each target and a score is generated based on the presumed number of copies within the targeted region. Targets with a score ≤ 0.5 (suspected of deletion) or ≥1.5 (suspected of duplication) are selected and subsequently confronted to data from the general population reported in CNV databases (e.g. Database of Genomic Variants, <http://dgv.tcag.ca/dgv/app/about>) to exclude common variants (>0.005 for recessive variants).
