# Supplementary material for: Identification of a Novel Homozygous Nonsense Mutation Confirms the Implication of GNAT1 in Rod-Cone Dystrophy
Source: PLoS One. 2016 Dec 15;11(12):e0168271. doi: 10.1371/journal.pone.0168271 (PMC5158031; doi:10.1371/journal.pone.0168271)
Supplement: S2 Methods — To highlight homozygous regions in rare recessive disorders, an in-house script, developed by a company (Integragen, Evry, France), was used from WES data. Indeed, the method takes advantage of the fact that affected individuals are likely to have two recessive copies of the disease allele from common ancestor alleles. (DOCX) [file pone.0168271.s002.docx]

**S2 Methods: Homozygosity mapping from WES data**

To highlight homozygous regions in rare recessive disorders, an in-house script, developed by a company (Integragen, Evry, France), was used from WES data. Indeed, the method takes advantage of the fact that affected individuals are likely to have two recessive copies of the disease allele from common ancestor alleles.
