# Supplementary material for: Identification of a Novel Homozygous Nonsense Mutation Confirms the Implication of GNAT1 in Rod-Cone Dystrophy
Source: PLoS One. 2016 Dec 15;11(12):e0168271. doi: 10.1371/journal.pone.0168271 (PMC5158031; doi:10.1371/journal.pone.0168271)
Supplement: S1 Table — (DOCX) [file pone.0168271.s003.docx]

**S1 Table: Coverage and read depth from whole exome sequencing for the affected boy, CIC01293.**

| Chr | Size of the target regions (bp) | 1X Coverage (%) | 4X Coverage (%) | 10X Coverage (%) | 25X Coverage (%) | Mean depth (X) |
| --- | --- | --- | --- | --- | --- | --- |
| 1 | 5 365 692 | 99 | 98 | 96 | 91 | 79 |
| 2 | 4 087 199 | 99 | 98 | 97 | 92 | 83 |
| 3 | 3 192 893 | 100 | 99 | 98 | 94 | 85 |
| 4 | 2 133 930 | 99 | 98 | 97 | 93 | 84 |
| 5 | 2 453 219 | 99 | 98 | 97 | 92 | 84 |
| 6 | 2 787 375 | 99 | 98 | 97 | 92 | 83 |
| 7 | 2 595 393 | 98 | 97 | 95 | 89 | 77 |
| 8 | 1 876 426 | 98 | 97 | 96 | 91 | 79 |
| 9 | 2 141 030 | 99 | 98 | 96 | 91 | 79 |
| 10 | 2 160 337 | 98 | 97 | 96 | 91 | 79 |
| 11 | 3 180 824 | 99 | 98 | 97 | 91 | 79 |
| 12 | 2 990 014 | 99 | 99 | 97 | 92 | 79 |
| 13 | 972 617 | 100 | 99 | 98 | 94 | 82 |
| 14 | 1 809 833 | 100 | 99 | 97 | 92 | 78 |
| 15 | 1 895 654 | 98 | 97 | 95 | 90 | 78 |
| 16 | 2 354 961 | 96 | 94 | 92 | 85 | 74 |
| 17 | 3 080 196 | 99 | 98 | 95 | 89 | 74 |
| 18 | 845 629 | 100 | 99 | 98 | 93 | 84 |
| 19 | 3 206 161 | 99 | 96 | 93 | 83 | 66 |
| 20 | 1 336 358 | 99 | 98 | 97 | 91 | 77 |
| 21 | 550 693 | 100 | 99 | 97 | 92 | 79 |
| 22 | 1 174 494 | 98 | 97 | 94 | 86 | 71 |
| X | 2 023 639 | 95 | 94 | 91 | 78 | 43 |
| Y | 114 128 | 54 | 52 | 49 | 40 | 23 |
| Total | 54 328 695 | 99 | 98 | 96 | 90 | 77 |
