# Supplementary material for: Identification of a Novel Homozygous Nonsense Mutation Confirms the Implication of GNAT1 in Rod-Cone Dystrophy
Source: PLoS One. 2016 Dec 15;11(12):e0168271. doi: 10.1371/journal.pone.0168271 (PMC5158031; doi:10.1371/journal.pone.0168271)
Supplement: S4 Table — 1Variant nomenclature was determined with a software (Alamut v2.4, Interactive Biosoftware, Rouen, France). 2Conservation: “Highly” means that the same amino acid is conserved in 100 species; “Moderately” means that the amino acid residue varies less than 5 times among species at this position but is conserved in primates; “Weakly” means the amino acid residue varies between 5 to 7 times; if the amino acid residue varies more than 7 times it is qualified as “Not conserved”. # means that the amino acid residue at the same position changes among primates, but not necessarily with the same amino acid change as the one found in the patient. 3ExAC gives the minor allele frequency in a large population from various ethnicities. “Unknown” means that the given change has not been reported in ExAC database. 4Retinal expression is determined using UniGene results (http://www.ncbi.nlm.nih.gov/unigene). 5Protein domains and splice effect predictions (Alamut v2.4, Interactive Biosoftware; MaxEntScan, http://genes.mit.edu/burgelab/maxent/Xmaxentscan_scoreseq.html, [33]; Splice Site Prediction by Neural Network, NNSPLICE, http://www.fruitfly.org/seq_tools/splice.html, [34]; Human Splicing Finder v.2.4.1, HSF, http://www.umd.be/HSF/#, [35]). G protein-coupled receptor, GPCR; seven-transmembrane domains, 7TM; immunoglobulin-like, plexins, transcription factors domain, IPT/TIG; flavin adenine dinucleotide, FAD. (DOCX) [file pone.0168271.s006.docx]

**S4 Table: Variant found homozygous in the affected boy, CIC01293.** ^1^Variant nomenclature was determined with a software (Alamut v2.4, Interactive Biosoftware, Rouen, France). ^2^Conservation: “Highly” means that the same amino acid is conserved in 100 species; “Moderately” means that the amino acid residue varies less than 5 times among species at this position but is conserved in primates; “Weakly” means the amino acid residue varies between 5 to 7 times; if the amino acid residue varies more than 7 times it is qualified as “Not conserved”. ^#^ means that the amino acid residue at the same position changes among primates, but not necessarily with the same amino acid change as the one found in the patient. ^3^ExAC gives the minor allele frequency in a large population from various ethnicities. “Unknown” means that the given change has not been reported in ExAC database. ^4^Retinal expression is determined using UniGene results (<http://www.ncbi.nlm.nih.gov/unigene>). ^5^Protein domains and splice effect predictions (Alamut v2.4, Interactive Biosoftware; MaxEntScan, <http://genes.mit.edu/burgelab/maxent/Xmaxentscan_scoreseq.html>, [[1](#_ENREF_1)]; Splice Site Prediction by Neural Network, NNSPLICE, <http://www.fruitfly.org/seq_tools/splice.html>, [[2](#_ENREF_2)]; Human Splicing Finder v.2.4.1, HSF, [http://www.umd.be/HSF/#](http://www.umd.be/HSF/), [[3](#_ENREF_3)]). G protein-coupled receptor, GPCR; seven-transmembrane domains, 7TM; immunoglobulin-like, plexins, transcription factors domain, IPT/TIG; flavin adenine dinucleotide, FAD.

| Chr | Gene name | Refseq and MIM# | Variant^1^ | Conservation^2^ | Minor allele frequency^3^ | PolyPhen2 | SIFT | Mutation Taster | Retinal expression^4^ | Comments^5^ |
| --- | --- | --- | --- | --- | --- | --- | --- | --- | --- | --- |
| 1 | *DVL1* | NM_004421.2  MIM *601365 | c.685C>G rs144365982 p.(Arg229Gly) | Not conserved | 0.0003480 | Benign | Tolerated | Disease causing | Yes | None |
| 1 | *PLCH2* | NM_014638.3  MIM *612836 | c.992C>T rs553813725 p.(Pro331Leu) | Moderately | 0.0000224 | Probably damaging | Deleterious | Disease causing | Yes | This variation is in protein domains: Phospholipase C, phosphatidylinositol-specific , X domain; Phosphoinositide phospholipase C |
| 1 | *STRIP1* | NM_033088.3 | c.596G>A p.(Cys199Tyr) | Highly | 0,0000109 | Possibly damaging | Deleterious | Disease causing | Yes | Predicted change at acceptor site 15 bps upstream: -5.0%; MaxEnt: 0.0%; NNSPLICE: -14.9%; HSF: 0.0%. This variation is in protein domain: N1221-like |
| 1 | *SLC16A4* | NM_004696.2  MIM *603878 | c.1043C>T rs141013255 p.(Thr348Met) | Not conserved^#^ | 0,0001403 | Possibly damaging | Deleterious | Disease causing | Yes | Predicted change at acceptor site 13 bps upstream: -4.5%; MaxEnt: 0.0%; NNSPLICE: -13.6%; HSF: 0.0%. This variation is in protein domains: Major facilitator superfamily; Major facilitator superfamily domain |
| 2 | *APOB* | NM_000384.2  MIM +107730 | c.35T>C rs758450840 p.(Leu12Pro) | Not conserved^#^ | Unknown | Benign | Deleterious | Polymorphism | No | None |
| 2 | *FN1* | NM_212482.1 MIM *135600 | c.5383G>A p.(Ala1795Thr) | Moderately | Unknown | Probably damaging | Deleterious | Disease causing | Yes | This variation is in protein domain: Fibronectin, type III. This variation is in a large homozygous region (35,9 Mb). |
| 2 | *MLPH* | NM_024101.6  MIM *606526 | c.1385T>C rs538560359 p.(L432Pro) | Highly | 0,0000888 | Probably damaging | Deleterious | Disease causing | Yes | This variation is in protein domain: Myelin-associated oligodendrocytic basic protein. This variation is in a large homozygous region (35,9 Mb). |
| **3** | ***GNAT1*** | **NM_144499.2**  **MIM *139330** | **c.923C>A p.(Cys321*)** | **Highly** | **0,0000083** | **-** | **-** | **-** | **Yes** | **Transversion from C to A in exon 8. Nonsense substitution. The reading frame is interrupted by a premature stop codon. This variation is in a large homozygous region (43,6 Mb).** |
| 3 | *IFRD2* | NM_006764.4  MIM *602725 | c.443T>G p.(Leu148Arg) | Not conserved | Unknown | Probably damaging | Deleterious | Disease causing | Yes | This variation is in protein domain: Interferon-related developmental regulator, N-terminal. This variation is in a large homozygous region (43,6 Mb). |
| 3 | *KALRN* | NM_001024660.3  MIM *604605 | c.2171C>T rs145790621 p.(Ser724Leu) | Not conserved | 0,0021762 | Benign | Tolerated | Disease causing | Yes | Predicted change at acceptor site 6 bps upstream: -1.6%; MaxEnt: 0.0%; NNSPLICE: -4.8%; HSF: 0.0%. This variation is in protein domain: Spectrin/alpha-actinin. This variation is in a large homozygous region (94,9 Mb). |
| 3 | *SLCO2A1* | NM_005630.2  MIM *601460 | c.1861C>T rs147416721 p.(Leu621Phe) | Moderately | 0,0000330 | Probably damaging | Tolerated | Disease causing | Yes | This variation is in protein domains: Organic anion transporter polypeptide OATP; Major facilitator superfamily domain. This variation is in a large homozygous region (94,9 Mb). |
| 3 | *HLTF* | NM_139048.2 | c.2411T>C rs61750365 p.(Ile804Thr) | Not conserved | 0,0027922 | Possibly damaging | Deleterious | Disease causing | Yes | None. This variation is in a large homozygous region (94,9 Mb). |
| 4 | *CHRNA9* | NM_017581.3  MIM *605116 | c.634A>G rs375870108 p.(Lys212Glu) | Not conserved | 0,0001071 | Probably damaging | Deleterious | Disease causing | No | This variation is in protein domains: Neurotransmitter-gated ion-channel ligand-binding; Neurotransmitter-gated ion-channel. This variation is in a large homozygous region (103,9 Mb). |
| 7 | *RELN* | NM_005045.3; MIM *600514 | c.7333C>G p.(Leu2445Val) | Not conserved | Unknown | Possibly damaging | Deleterious | Disease causing | Yes | None. This variation is in a large homozygous region (37,2 Mb). |
| 7 | *SLC13A1* | NM_022444.3; MIM *606193 | c.34C>T rs28364172 p.(Arg12*) | Moderately | 0,0020132 | - | - | - | No | Transition from C to T in exon 1. Nonsense substitution. The reading frame is interrupted by a premature stop codon. The mRNA produced might be targeted for nonsense mediated decay (NMD). This variation is in a large homozygous region (37,2 Mb). |
| 7 | *GPR37* | NM_005302.3  MIM *602583 | c.1081C>A p.(Arg361Ser) | Moderately | Unknown | Probably damaging | Deleterious | Disease causing | Yes | This variation is in protein domains: GPCR, rhodopsin-like, 7TM; GPCR, rhodopsin-like superfamily. This variation is in a large homozygous region (37,2 Mb). |
| 7 | *CPA4* | NM_016352.3  MIM *607635 | c.777G>A rs145012020 p.(Trp259*) | Moderately | 0,0023494 | - | - | - | Yes | Transition from G to A in exon 8. Nonsense substitution. The reading frame is interrupted by a premature stop codon. The mRNA produced might be targeted for nonsense mediated decay (NMD). This variation is in a large homozygous region (37,2 Mb). |
| 7 | *PLXNA4* | NM_020911.1  MIM *604280 | c.2875C>A p.(Leu959Met) | Moderately | Unknown | Probably damaging | Deleterious | Disease causing | Yes | Predicted change at acceptor site 19 bps upstream: +2.1%; MaxEnt: +0.0%; NNSPLICE: +6.2%; HSF: +0.0%. This variation is in protein domain: Cell surface receptor IPT/TIG. This variation is in a large homozygous region (37,2 Mb). |
| 8 | *HNF4G* | NM_004133.4  MIM *605966 | c.1173A>G p.(Ile391Met) | Not conserved | Unknown | Benign | Tolerated | Disease causing | No | None. This variation is in a large homozygous region (38,7 Mb). |
| 9 | *CCDC171* | NM_173550.2 | c.1261G>A rs139248677 p.(Val421Met) | Not conserved | 0,0006754 | Benign | Deleterious | Polymorphism | No | None |
| 9 | *FOCAD* | NM_017794.4  MIM *614606 | c.2336C>T rs147376982 p.(Thr779Ile) | Not conserved | 0,0012934 | Benign | Deleterious | Disease causing | Yes | None |
| 9 | *TMEM2* | NM_013390.2  MIM *605835 | c.4919A>T rs146439095 p.(Asp164Val) | Highly | 0,0024328 | Probably damaging | Deleterious | Disease causing | Yes | Predicted change at acceptor site 19 bps upstream: +3.1%; MaxEnt: +0.0%; NNSPLICE: +9.2%; HSF: +0.0%. This variation is in protein domain: G8 domain |
| 9 | *PRUNE2* | NM_015225.2  MIM *610691 | c.1454G>T p.(Gly485Val) | Not conserved | Unknown | Probably damaging | Deleterious | Disease causing | Yes | None |
| 10 | *NPFFR1* | NM_022146.4  MIM *607448 | c.8-2A>G rs199731225 p.(?) | Only nucleotide sequences | 0,0028815 | - | - | - | No | Predicted change at acceptor site 2 bps downstream: -100.0%; MaxEnt: -100.0%; NNSPLICE: -100.0%; HSF: -100.0%. This variation is in a large homozygous region (31,9 Mb). |
| 11 | *ZNF215* | NM_013250.2  MIM *605016 | c.712+1G>A rs138660620 p.(?) | Only nucleotide sequences | 0,0012682 | - | - | - | Yes | Predicted change at donor site 1 bps upstream: -100.0%; MaxEnt: -100.0%; NNSPLICE: -100.0%; HSF: -100.0% |
| 11 | *NELL1* | NM_001288713.1  MIM *602319 | c.1748A>C rs201683742 p.(Glu583Ala) | Moderately | 0,0002804 | Possibly damaging | Deleterious | Disease causing | No | Predicted change at acceptor site 19 bps upstream: +3.1%; MaxEnt: +0.0%; NNSPLICE: +9.2%; HSF: +0.0% |
| 11 | *OR4A15* | NM_001005275.1 | c.917T>A p.(Ile306Lys) | Not conserved^#^ | 0,0000082 | Probably damaging | Deleterious | Polymorphism | Unknown | This variation is in protein domains: GPCR, rhodopsin-like, 7TM; GPCR, rhodopsin-like superfamily |
| 12 | *RERGL* | NM_024730.3 | c.583A>G p.(Asn195Asp) | Not conserved^#^ | Unknown | Probably damaging | Tolerated | Disease causing | No | None. This variation is in a large homozygous region (39 Mb). |
| 12 | *MPHOSPH9* | NM_022782.3  MIM *605501 | c.1339A>G rs147793766 p.(Thr447Ala) | Not conserved^#^ | 0,0029753 | - | Deleterious | - | Yes | None |
| 15 | *DUOX2* | NM_014080.4  MIM *606759 | c.3910C>T rs534696164 p.(Arg1304Trp) | Highly | 0,0000742 | Probably damaging | Deleterious | Disease causing | Yes | This variation is in protein domain: FAD-binding 8. This variation is in a large homozygous region (39,6 Mb). |
| 15 | *RFX7* | NM_022841.5  MIM *612660 | c.1753G>A rs140370063 p.(Glu585Lys) | Not conserved | 0,0001639 | Possibly damaging | Tolerated | Disease causing | Yes | None. This variation is in a large homozygous region (39,6 Mb). |
| 15 | *UBAP1L* | NM_001163692.1 | c.121-5_126del p.(?) | Moderately | Unknown | - | - | - | Yes | Predicted change at acceptor site 0 bps upstream: -6.3%; MaxEnt: -19.6%; NNSPLICE: -0.1%; HSF: 0.9%. This variation is in a large homozygous region (39,6 Mb). |
| 15 | *CHRNA5* | NM_00745.3  MIM *118505 | c.43C>T p.(Leu15Ile) | Only nucleotide sequences | Unknown | Benign | Deleterious | Polymorphism | Yes | None |
| 20 | *ADAM33* | NM_025220.4  MIM *607114 | c.1184C>T rs3918394 p.(Ala395Val) | Not conserved | 0,003221 | Possibly damaging | Tolerated | - | Yes | This variation is in protein domain: Peptidase M12B, ADAM/reprolysin |
| X | *DMD* | NM_004006.2  MIM *300377 | c.2971G>C rs72468667 p.(Glu991Gln) | Weakly | 0,0015396 | Probably damaging | Deleterious | Polymorphism | Yes | This variation is in protein domains:  Spectrin repeat; Spectrin/alpha-actinin; Dystrophin/utrophin. This variation is in a large homozygous region (51,9 Mb). |

1. Yeo G, Burge CB (2004) Maximum entropy modeling of short sequence motifs with applications to RNA splicing signals. J Comput Biol 11: 377-394.

2. Reese MG, Eeckman FH, Kulp D, Haussler D (1997) Improved splice site detection in Genie. J Comput Biol 4: 311-323.

3. Desmet FO, Hamroun D, Lalande M, Collod-Beroud G, Claustres M, et al. (2009) Human Splicing Finder: an online bioinformatics tool to predict splicing signals. Nucleic Acids Res 37: e67.
