# Supplementary material for: Identification of a Novel Homozygous Nonsense Mutation Confirms the Implication of GNAT1 in Rod-Cone Dystrophy
Source: PLoS One. 2016 Dec 15;11(12):e0168271. doi: 10.1371/journal.pone.0168271 (PMC5158031; doi:10.1371/journal.pone.0168271)
Supplement: S5 Table — 1Variant nomenclature was determined with a software (Alamut v2.4, Interactive Biosoftware, Rouen, France). 2Conservation: “Highly” means that the same amino acid is conserved in 100 species; “Moderately” means that the amino acid residue varies less than 5 times among species at this position but is conserved in primates; “Weakly” means the amino acid residue varies between 5 to 7 times; if the amino acid residue varies more than 7 times it is qualified as “Not conserved”. # means that the amino acid residue at the same position changes among primates, but not necessarily with the same amino acid change as the one found in the patient. 3ExAC gives the minor allele frequency in a large population from various ethnicities. “Unknown” means that the given change has not been reported in ExAC database. 4Retinal expression is determined using UniGene results (http://www.ncbi.nlm.nih.gov/unigene). 5Protein domains and splice effect predictions (Alamut v2.4, Interactive Biosoftware; MaxEntScan, http://genes.mit.edu/burgelab/maxent/Xmaxentscan_scoreseq.html, [33]; Splice Site Prediction by Neural Network, NNSPLICE, http://www.fruitfly.org/seq_tools/splice.html, [34]; Human Splicing Finder v.2.4.1, HSF, http://www.umd.be/HSF/#, [35]). (DOCX) [file pone.0168271.s007.docx]

**S5 Table: Variant found heterozygous in the affected boy, CIC01293.** ^1^Variant nomenclature was determined with a software (Alamut v2.4, Interactive Biosoftware, Rouen, France). ^2^Conservation: “Highly” means that the same amino acid is conserved in 100 species; “Moderately” means that the amino acid residue varies less than 5 times among species at this position but is conserved in primates; “Weakly” means the amino acid residue varies between 5 to 7 times; if the amino acid residue varies more than 7 times it is qualified as “Not conserved”. ^#^ means that the amino acid residue at the same position changes among primates, but not necessarily with the same amino acid change as the one found in the patient. ^3^ExAC gives the minor allele frequency in a large population from various ethnicities. “Unknown” means that the given change has not been reported in ExAC database. ^4^Retinal expression is determined using UniGene results (<http://www.ncbi.nlm.nih.gov/unigene>). ^5^Protein domains and splice effect predictions (Alamut v2.4, Interactive Biosoftware; MaxEntScan, <http://genes.mit.edu/burgelab/maxent/Xmaxentscan_scoreseq.html>, [[1](#_ENREF_1)]; Splice Site Prediction by Neural Network, NNSPLICE, <http://www.fruitfly.org/seq_tools/splice.html>, [[2](#_ENREF_2)]; Human Splicing Finder v.2.4.1, HSF, [http://www.umd.be/HSF/#](http://www.umd.be/HSF/), [[3](#_ENREF_3)]).

| Chr | Gene name | Refseq and MIM# | Variant^1^ | Conservation^2^ | Minor allele frequency^3^ | PolyPhen2 | SIFT | Mutation Taster | Retinal expression^4^ | Comments^5^ |
| --- | --- | --- | --- | --- | --- | --- | --- | --- | --- | --- |
| 8 | *PLEC* | NM_201380.2  MIM *601282 | c.9445G>A rs200898220 p.(Glu3149Lys) | Not conserved | 0,0010226 | Benign | Deleterious | - | Yes | This variation is in protein domain: Plectin repeat |
|  |  |  | c.2800C>T p.(Arg934Cys) | Moderately | 0,0000306 | Probably damaging | Deleterious | - |  | This variation is in protein domain: Spectrin/alpha-actinin |
| 14 | *SLC25A29* | NM_001039355.2  MIM *615064 | c.442C>T rs148590600 p.(Arg148Cys) | Not conserved | 0,0026618 | Probably damaging | Deleterious | Disease causing | Yes | This variation is in protein domain: Mitochondrial substrate/solute carrier |
|  |  |  | c.213T>G p.(Phe71Cys) | Highly | Unknown | Probably damaging | Deleterious | Disease causing |  | This variation is in protein domain: Mitochondrial substrate/solute carrier |
| 15 | *VPS33B* | NM_018668.3  MIM *608552 | c.1236del p.(Lys413Argfs*6) | Highly | Unknown | - | - | - | Yes | Deletion (1 bp) in exon 17. This deletion creates a frame shift starting at codon Lys413. The new reading frame ends in a stop codon 5 positions downstream. The mRNA produced might be targeted for nonsense mediated decay (NMD). |
|  |  |  | c.1235C>G p.(Pro412Arg) | Not conserved^#^ | 0,0000495 | Possibly damaging | Tolerated | - |  | None |
| 17 | *WSCD1* | NM_015253.1 | c.329G>A p.(Arg110His) | Moderately | 0,0000000 | Probably damaging | Deleterious | Disease causing | Yes | None |
|  |  |  | c.1609G>A rs138045353 p.(Asp537Asn) | Not conserved | 0,0008468 | Benign | Tolerated | Disease causing |  | None |
| 20 | *FOXS1* | NM_004118.3  MIM *602939 | c.559C>T rs149428291 p.(Arg187Trp) | Not conserved^#^ | 0,0010377 | Probably damaging | Deleterious | Polymorphism | No | None |
|  |  |  | c.220G>A rs45499294  p.(Glu74Lys) | Weakly | 0,0024722 | Probably damaging | Deleterious | Disease causing |  | This variation is in protein domain: Transcription factor, fork head |

1. Yeo G, Burge CB (2004) Maximum entropy modeling of short sequence motifs with applications to RNA splicing signals. J Comput Biol 11: 377-394.

2. Reese MG, Eeckman FH, Kulp D, Haussler D (1997) Improved splice site detection in Genie. J Comput Biol 4: 311-323.

3. Desmet FO, Hamroun D, Lalande M, Collod-Beroud G, Claustres M, et al. (2009) Human Splicing Finder: an online bioinformatics tool to predict splicing signals. Nucleic Acids Res 37: e67.
