# Supplementary material for: Identification of a Novel Homozygous Nonsense Mutation Confirms the Implication of GNAT1 in Rod-Cone Dystrophy
Source: PLoS One. 2016 Dec 15;11(12):e0168271. doi: 10.1371/journal.pone.0168271 (PMC5158031; doi:10.1371/journal.pone.0168271)
Supplement: S6 Table — (DOCX) [file pone.0168271.s008.docx]

**S6 Table: Genotype of the different family members for three genetic markers.**

| Chr | Position | rs | CIC01293 affected boy's genotype | CIC01294 unaffected father's genotype | CIC06690 unaffected mother's genotype |
| --- | --- | --- | --- | --- | --- |
| 12 | 21015760 | rs7311358 | GG | AA | GA |
| 12 | 125284748 | rs5888 | AA | GG | AG |
| 22 | 19951271 | rs4680 | AA | GG | GA |
| Y | 4968368 | rs2524543 | TT | GG | / |
| Y | 5491165 | rs766378099 | CC | AA | / |
| Y | 15581983 | rs2032658 | AA | GG | / |
