# Supplementary material for: Identification of a Novel Homozygous Nonsense Mutation Confirms the Implication of GNAT1 in Rod-Cone Dystrophy
Source: PLoS One. 2016 Dec 15;11(12):e0168271. doi: 10.1371/journal.pone.0168271 (PMC5158031; doi:10.1371/journal.pone.0168271)
Supplement: S7 Table — Bold large homozygous region includes GNAT1 variant c.923C>A p.(Cys321*). (DOCX) [file pone.0168271.s009.docx]

**S7 Table: Large (>30 Mb) homozygous regions found in the affected boy.** Bold large homozygous region includes *GNAT1* variant c.923C>A p.(Cys321*).

| Chrom | Start | End | Length (bp) | Length (Mb) |
| --- | --- | --- | --- | --- |
| 2 | 203155936 | 239007555 | 35851619 | 35,9 |
| **3** | **32031962** | **75679938** | **43647976** | **43,6** |
| 3 | 75986717 | 170857240 | 94870523 | 94,9 |
| 4 | 16227956 | 120133549 | 103905593 | 103,9 |
| 5 | 10282396 | 73144845 | 62862449 | 62,9 |
| 7 | 27807450 | 72413581 | 44606131 | 44,6 |
| 7 | 100647676 | 137801413 | 37153737 | 37,2 |
| 8 | 62838152 | 101586133 | 38747981 | 38,7 |
| 10 | 8092806 | 38894600 | 30801794 | 30,8 |
| 10 | 47087501 | 78977190 | 31889689 | 31,9 |
| 12 | 11506669 | 50467769 | 38961100 | 39,0 |
| 13 | 61109375 | 108518727 | 47409352 | 47,4 |
| 15 | 29009311 | 68649587 | 39640276 | 39,6 |
| 18 | 14543092 | 76740298 | 62197206 | 62,2 |
| X | 3239979 | 55172630 | 51932651 | 51,9 |
| X | 55172708 | 135956370 | 80783662 | 80,8 |
